# Veno-arterial extracorporeal membrane oxygenation for electrical injury induced cardiogenic shock support: a case report

**DOI:** 10.1186/s13019-020-01188-x

**Published:** 2020-06-17

**Authors:** Tamer Jamal, Amjad Shalabi, Liza Grosman-Rimon, Diab Ghanim, Offer Amir, Erez Kachel

**Affiliations:** 1 Cardiac Surgery Department, B Padeh Medical Center, Poriya, Israel; 2 Department of Cardiac Surgery, Sheba Medical Centre, 5265601 Tel Hashomer, Israel; 3 The Azrieli Faculty of Medicine in the Galilee, Bar-Ilan University, Safed, Israel

**Keywords:** Extracorporeal membrane oxygenation, Cardiogenic shock support, Electrical injury: case report

## Abstract

**Background:**

High voltage electrical injury (HVEI) of more than 1000 V is a potentially devastating form of a multisystem injury associated with high morbidity and mortality.

We present the first case of veno-arterial extracorporeal membrane oxygenation (VA-ECMO) as a life saving device for treating a patient with severe cardiogenic shock after a high voltage electrical injury.

**Case presentation:**

A 26-year-old male sustained HVEI while working with a concrete mixer pump that came in contact with a high voltage cable of 10,000 V. He was immediately disconnected from the mixer pump, underwent cardiopulmonary resuscitation and was transported to the nearest medical centre with severe cardiogenic shock with an ejection fraction (EF) of < 10%.

Upon arrival, he was in critical condition, sedated and mechanically ventilated, haemodynamically unstable and supported by intravenous (IV) inotropes after a few events of ventricular fibrillation, with an electrical entry point on the left hand and an exit point located on his right leg. Blood pH was 6.8, PCO_2_ 53 mmHg, PaO_2_ of 57 mmHg, lactate 8 mmol/L, and Troponin 38,000 ng/dl. The EF was 10% with global severe left ventricular dysfunction. During cardiopulmonary resuscitation (CPR), including cardiac massage and few electrical shocks, he was immediately connected to the VA-ECMO via open right femoral approach with distal arterial leg perfusion.

He was treated with IV broad spectrum antibiotics, and high volume fluids to prevent rhabdomyolysis-induced acute kidney injury, total parenteral nutrition, topical silver sulfadiazine cream, and Granuflex for severe electrical burns. He was gradually weaned from inotropes over the next 3 days, during which his clinical condition and bloodwork improved tremendously. His EF gradually increased to 50% and he was weaned from the VA-ECMO, and underwent decannulation 86 h after initialization. He was discharged on day 27 without any sequelae.

**Conclusion:**

The VA-ECMO treatment can be a lifesaving device for treating severe cardiogenic shock caused by high voltage electrical injury, and should be considered while treating these “high-mortality risk” patients.

## Introduction

Electrical injury is an infrequent but potentially devastating form of multisystem injury associated with high morbidity and mortality [1]. Despite significant improvement in injury prevention and implementation of safety protocols at work places, electrical injury accounts for more than 500 deaths per year in the United States with a mortality rate of 10–30% [2]. Electrical injuries are divided into low-voltage electric power injuries (less than 1000 V) and high-voltage electric power injuries (more than 1000 V) [2].

Cardiac injuries induced by electrical shock can be divided into arrhythmias, conduction abnormalities, and myocardial damage – whether there is direct electricity exposure or secondary myocardial injury after hypotension, or coronary arteries spasm [3–5]. Early treatment of cardiogenic shock with appropriate circulation support may be vital with veno-arterial extracorporeal membrane oxygenation (VA-ECMO), as one of the therapeutic armamentarium in refractory cardiogenic shock [6].

Although veno-venous- ECMO (VV-ECMO) was used previously to support lung failure after electrical injury [7], the VA-ECMO to support cardiogenic shock secondary to high voltage electrical injury, has not yet been described. We present the first case demonstrating the role of VA-ECMO in resuscitating electrically injured patients.

## Case presentation

A 26-year-old man sustained a high voltage electrical injury (> 1000 V) while he was working with a concrete mixer pump that came in contact with high voltage cable of 10,000 V. The patient was disconnected from the tube immediately due to the jolt, lost consciousness and a cardiac massage was launched within 3 min by witnesses. Then, advanced life support was supplied by paramedics as ventricular fibrillation was detected. He was referred to the nearby emergency room (ER) of a secondary hospital with cardiogenic shock, being chest compressed by a LUCAS machine. In the ER, advanced cardiovascular life support continued for more than 30 min.

After resuscitation, the patient was still supported with IV Noradrenaline. He was then referred to our institute. A physical exam conducted upon his admission showed an unconscious ventilated patient with reactive dilated pupils, tachycardia and a normal bilateral vesicular breathing sound. Electrical entry point was detected on the left hand; exit points were on the feet, bilaterally. ECGs showed sinus tachycardia with PVCs. The arterial blood test showed: PH 6.8, PCO_2_ 53mmHG, PaO_2_ 57 mmHg, lactate 8 mmol/l, and troponin 38,000 ng/dl. The ECG showed an estimated ejection fraction (EF) of 10%, with global dysfunction and a reserved valves’ function. After the heart team consulted, the patient was then connected to the VA-ECMO within 30 min of his admission, via a right femoral approach with distal perfusion.

### Management

VA-ECMO support was supplied for 86 h (3.6 days).

During this period, multiple signs and blood tests were performed, among them those related to VA-ECMO (Tables 1 and 2).

**Table 1:** Patient’s Clinical Outcomes

Clinical Outcomes	
Flow	4 L/MIN
Round per minute	3360
Pre-membranous pressure	200
Post-membranous pressure	170
ECMO FIO2	60%
ACT	180–220
BRAIN SAT	70%
PUPIL EXAM	ROUND, REACTIVE TO LIGHT
LOWER LIMB SAT	RIGHT 90%, LEFT 70%

**Table 2:** Patient’s Biomarker Levels Before, During cannulationafter ECMO

	Day 1 of admission	After 2 days on ECMO	1 day after ECMO decannulation	ARDS and referral day
**WBC** (uL/1000)	36.6	18	19	25
HEMOGLOBIN (g/dl)	18	10	9.9	9.6
**PLATELETS** (uL/1000)	343	167	150	950
**CREATININE** (mg/dl)	1.5	1.1	1	2.5
**LDH** (U/L)	1315	1650	950	950
**CPK** (U/L)	8250	18000	10000	2800
**AST** (U/L)	1200	450	450	270
**ALT** (U/L)	425	150	150	130
**LACTATE** (mmol/l)	8	0.6	1.4	1.9
**PH**	6.8	7.45	7.4	7.32

Other points of care included: suspicion of aspiration on the day of admission; he was treated by antibiotics. Diagnosis of right pleural effusion which was drained by a chest tube. Fluid administration to prevent rhabdomyolysis induced acute kidney injury. Total parenteral nutrition for visceral electrical injury, which resulted in decreased enteral absorption. Burn wounds were treated locally by utilizing silver sulfadiazine cream (Silverol) and Granuflex dressings.

### Outcomes

Within the first day, the patient had been weaned from noradrenaline. The lactate level declined gradually down to 0.6 mmol/L. A follow up echocardiography on the third day showed improvement in cardiac function, with an EF = 50% and normal right ventricular function. After 86 h of mechanical support, the patient underwent decannulation from VA-ECMO successfully.

The weaning process was gradual, starting with decreasing the VA-ECMO to 1 l/min for 24 h before decannulation, during this period a trans oesophageal echocardiography was completed, making sure biventricular functions were within the normal range and there was no ventricular dilatation. Chest X-rays showed no new opacities or indications of ARDS. Other parameters that we followed within this period were Central Venous Pressure (CVP), PH, lactate and target organ blood test, to endure normal function.

On the day of decannulation, we stopped heparin, once ACT was less than 160, we conducted decannulation successfully. A transthoracic echocardiography conducted 1 day after the decannulation showed normal biventricular and valves function.

**Fig. 1:**
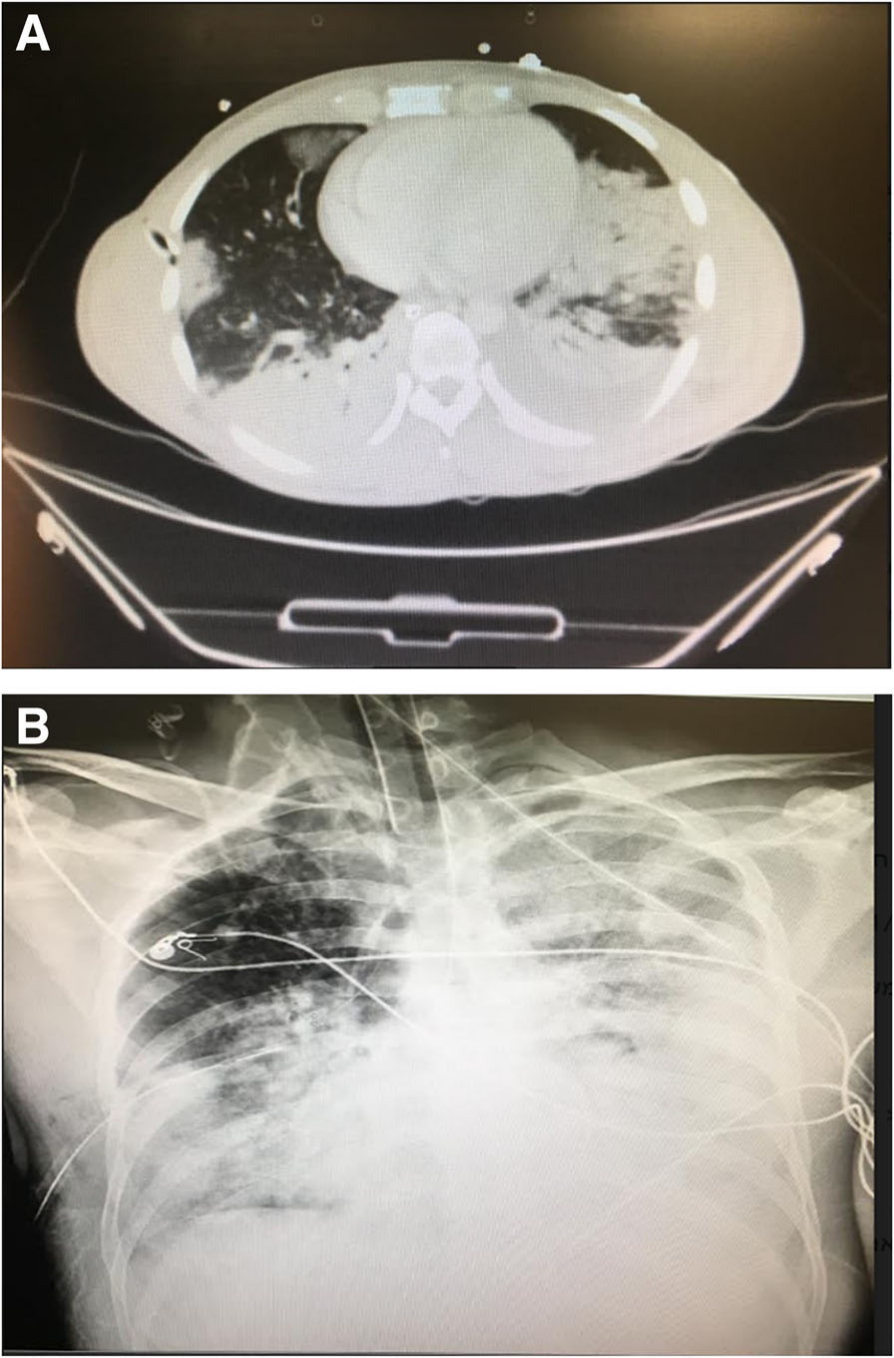
**a** Computed tomogram of the patient with ARDS, demonstrating bilateral dependent consolidation, a large consolidation in the LUL, and small peripheral patchy alveolar infiltrate on the right. **b** A chest radiograph of the patient on the 6th day of hospitalization, showing ARDS with bilateral infiltrates and bilateral heterogeneous consolidations, more on the left side

Acute respiratory distress syndrome (ARDS) was diagnosed on the 6th day of hospitalization and was treated accordingly Fig. 1.

On the seventh day, the patient was referred to the ARDS specialized department in a primary medical centre, being anesthetised and ventilated for continuous treatment. There, he was hospitalized for almost a month. During this period he passed tracheostomy and then was gradual weaned and underwent respiratory physiotherapy up until discharge. No plastic surgery was done and the burns were treated topically. Two months after the accident, he was discharged and moved to rehabilitation centre.

**Fig. 2:**
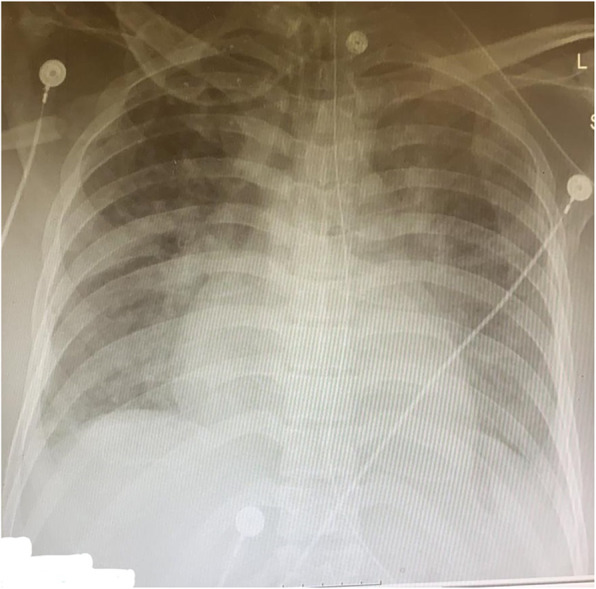
Chest x-ray at discharge from the ARDS specialized department, showing no infiltrates or consolidation

His chest X-rays at the discharge from the ARDS specialized department is shown in Fig. 2. His blood tests at discharge were normal, especially renal functions – creatinine 0.7 mg/dl.

A follow-up transthoracic echocardiography showed – left ventricle with normal global systolic function (EF = 65%) with no regional wall abnormality. The right ventricle was found to be normal size and properly functioning. The aortic valve was normally functioning, as were the mitral, tricuspid and pulmonary valve, with minimal regurgitation.

## Discussion

The patient presented with high voltage electrical burn induced cardiogenic shock, with multi-organ failure; instant transthoracic echocardiography indicated EF of 10%,so he was immediately supported by the VA-ECMO.

To our knowledge, veno-venous- ECMO (VV-ECMO) was used to support lung failure after electrical injury [7]. However, this is the first report of cardiogenic shock requiring VA-ECMO support, secondary to high voltage electrical injury. For note, VA-ECMO treatments may not be effective in all high voltage electrical injury cases.

The rationales for VA-ECMO connection in our case were the young age, low cardiac output in the setting of multiple organ dysfunction, and the risk of additional arrhythmias that might deteriorate cardiac function further and lead to dreadful results. So, we believed that the circulatory support via VA-ECMO will supply better perfusion, even in the presence of serious arrhythmias, for the target organs and improving their function. Moreover, we took into consideration that VA-ECMO can be used as a bridge for surgical salvage (assistant device implantation or cardiac transplant) in the case of persistent heart failure.

Cardiac injury after an electrical jolt may happen due to arrhythmias - ventricular fibrillation can occur with low-voltage alternating current, whereas asystole is more common with direct current or high-voltage alternating current [3]. The mechanism behind electrically induced cardiac arrhythmias is not entirely clear but may involve patchy areas of myocardial necrosis, which serve as arrhythmogenic foci, as well as increased cardiac sodium-potassium pump activity [4].

Moreover, electrical exposure may cause direct myocardial tissue damage through transcardiac passage of the electric current or indirect damage through ischemic injury, precipitated by arrhythmia-induced hypotension or a coronary artery spasm [3].

Electrical energy is capable of damaging most organs, depending on current intensity and pathway, which could lead to a life-threatening clinical state [5]. Organ damage by electrical energy includes superficial and deep burn wounds, rhabdomyolysis/ compartment syndrome, acute renal failure due to myoglobinurea or ischemia as well as respiratory arrest, as a result of either direct injury to the respiratory control centre, causing cessation of respiration, or to suffocation secondary to tetanic contractions of the respiratory muscles [5].

The recovery of these organs’ functions is challenging, especially in cases of a failing heart. We think that in such electrically injured moribund patients, VA-ECMO can be an instant salvage for mechanical circulatory support, and can help in restoring organ function, including the heart. In our case we used VA-ECMO to stabilize the patient instantly and to supply effective support for the organs until the heart regained its own normal function.

## Conclusion

VA-ECMO can be a crucial lifesaving device for treating severe cardiogenic shock caused by electrical injury, especially in high voltage electrical injury, and should be considered while treating these high-mortality risk patients.

## Data Availability

Please contact the authors for data requests.

## Abbreviations

HVEI: High voltage electrical injury
EF: Ejection fraction
VA-ECMO: Veno-arterial extracorporeal membrane oxygenation
IV: Intravenous
CPR: Cardiopulmonary resuscitation
ER: Emergency room
VV-ECMO: Veno-venous-extracorporeal membrane oxygenation
ARDS: Acute respiratory distress syndrome
